# The first documented volcanic eruption of Hayli Gubbi, Afar, Ethiopia

**DOI:** 10.1007/s00445-026-01997-3

**Published:** 2026-06-30

**Authors:** Dereje Ayalew, Karen Fontijn, Faysel Sefa Abdu, Derek Keir, Amdemichael Z. Tadesse, Carolina Pagli, Alessandro La Rosa, Pablo Tierz, Abate Melaku, William Hutchison, Patrick Sugden, Gezahegn Yirgu, Asfie M. Nigussie, Osman Ahmed, Hindeya Gebru, Martin F. Mangler, Matthew J. Cooper, Atalay Ayele

**Affiliations:** 1 School of Earth Sciences, Addis Ababa University, Addis Ababa, Ethiopia; 2 Department of Geosciences Environment and Society, Université libre de Bruxelles, Brussels, Belgium; 3 School of Ocean and Earth Science, University of Southampton, Southampton, UK; 4 Department of Earth Sciences, University of Florence, Florence, Italy; 5 Lamont Doherty Earth Observatory, University of Columbia, NY, USA; 6 Department of Earth Sciences, University of Pisa, Pisa, Italy; 7 Geociencias Barcelona (GEO3BCN), CSIC, Barcelona, Spain; 8 School of Earth and Environmental Sciences, University of St., Andrews, St. Andrews, UK; 9 SORA Tours Ethiopia, Addis Ababa, Ethiopia; 10 School of Earth Sciences, Samara University, Samara, Ethiopia; 11 Institute of Geophysics Space Science and Astronomy, Addis Ababa University, Addis Ababa, Ethiopia

**Keywords:** Explosive eruption, Hayli Gubbi, Ethiopia, Afar, East African Rift

## Abstract

Hayli Gubbi volcano in the Afar Regional State of Ethiopia experienced its first historically documented volcanic eruption on 23 November 2025. The event produced a short-lived sustained eruption column, with ash dispersal and fallout primarily towards the north and east of the volcano, and the formation of two new craters at Hayli Gubbi’s summit. Ash from the northern lobe has a basaltic (trachy)andesitic composition. The eruption was preceded, in July 2025, by a dyke intrusion originating from neighbouring Erta Ale volcano, migrating over 30 km southward and intercepting Hayli Gubbi where inflation occurred. Renewed ground deformation started again in Erta Ale ~ one month before the Hayli Gubbi eruption during which a sudden reservoir contraction and a new collapse caldera was formed. Synchronous deformation signals suggest both volcanoes are structurally coupled. Intense degassing continues at Hayli Gubbi during the months after the eruption. We provide the first accounts of the eruption’s development, assembling on-the-ground observations, geophysical and geochemical data and satellite imagery to characterize the physical features of the eruption and its impact. Whilst impact remained limited, partly due to low exposure in the remote region, the eruption did cause disruption to international air traffic. This eruption is another stark reminder of the urgent need for increased capacity for volcano monitoring and data-supported volcanic hazard assessment in Ethiopia.

**Supplementary Information:**

The online version contains supplementary material available at 10.1007/s00445-026-01997-3.

## Introduction

**Fig. 1:**
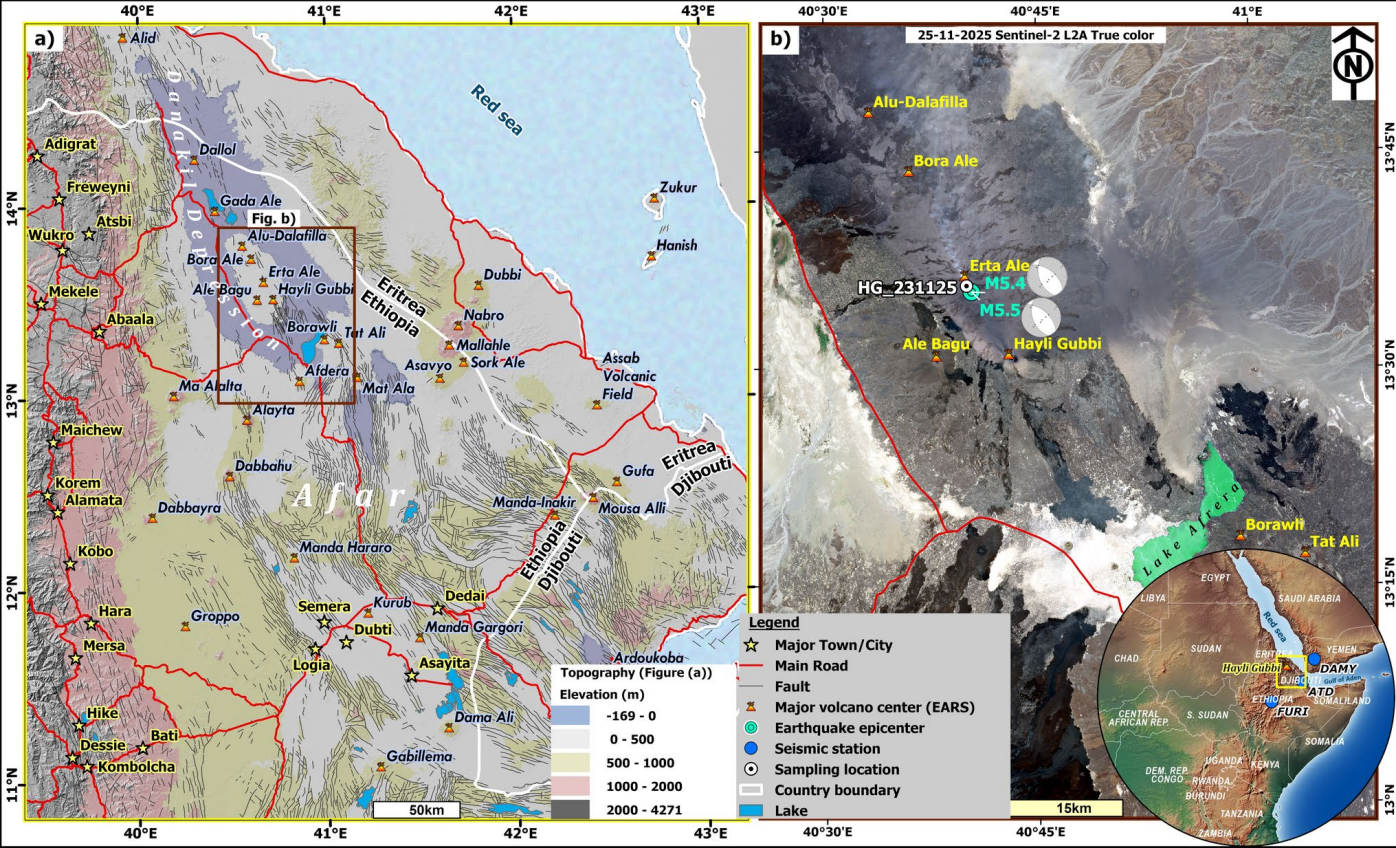
**a** Geographic setting of the Afar volcanic depression, with Holocene active volcanic systems (Global Volcanism Program 2025), international borders and main roads and settlements. **b** True-colour Sentinel-2 image covering the central and southern sections of the Erta Ale Range including Hayli Gubbi; rough location of two Mw 5.4 and 5.5 earthquakes around midday (UTC) of 23 November 2025 is indicated, as well as ash sampling location ~ 10 km North of Hayli Gubbi, on the western flank of Erta Ale

The volcanoes of the Afar Depression (Fig. 1) in northern Ethiopia, Eritrea and Djibouti have mostly been studied for their historical dyke intrusion episodes (e.g. Dabbahu–Manda Hararo; Ferguson et al. 2010) and basaltic effusive activity (e.g. Erta Ale; Field et al. 2012). Most volcanoes in Afar are for this reason considered dominated by effusive activity, and mostly erupting magmas of basaltic composition. Historically recorded explosive eruptions at Afar volcanoes are rare, and the two most significant ones both occurred at the Nabro Volcanic Range near the Ethiopia–Eritrea border. The most recent, and only historically recorded, eruption at Nabro occurred on 12 June 2011 and lasted several days. This eruption emplaced an 18 km^2^ lava flow and deposited tephra of trachybasaltic to trachybasaltic-andesite composition up to at least 10 km to the west and south of the volcano. Despite this being a relatively mafic eruption within the Nabro suite (Donovan et al. 2018), the combined volume of lava flow and tephra deposits yields an estimated magnitude of 5.1 (Goitom et al. 2015). The largest historical effusive-explosive eruption known in Afar occurred at Dubbi volcano (Eritrea) in 1861. This event emplaced a large basaltic lava flow and extensive trachytic pyroclastic density current (PDC) deposits up to 25 km from source, covered by co-ignimbrite ash fall (Wiart and Oppenheimer 2000; Wiart et al. 2000).

The potential for explosive activity at Afar volcanoes is not fully known. In this paper, we provide a brief description and geophysical and geochemical observations of the first historically documented (explosive) eruption at Hayli Gubbi, on the southern end of the Erta Ale Volcanic Range (Fig. 1). The eruption was short-lived and occurred on 23 November 2025, after an extended episode of multiple months of minor-moderate ground deformation due to dyke and sill inflation. The absence of a dedicated ground-based monitoring system in the region meant that the eruption occurred without much immediate detected precursors. Whilst impact remained limited, partly due to low exposure in the remote region, this eruption is a stark reminder of the urgent need for increased capacity for volcano monitoring and data-supported volcanic hazard assessment in Ethiopia (Tierz et al. 2020; Lewi et al. 2025).

## Geological setting

Hayli Gubbi volcano forms the southern part of the Erta Ale Volcanic Range, the most active axial volcanic segment in the Danakil Depression in the Afar region (Fig. 1). Magmatism in this region has been primarily attributed to a combination of mantle plume and rift-related decompression melting (Ferguson et al. 2013; Watts et al. 2025). The extension across the Danakil Depression is directed ~ NE-SW, at a rate of ~ 14–18 mm/yr (Viltres et al. 2020; Hurman et al. 2025). Erta Ale itself is most known for its semipermanent summit lava lake that has repeatedly overflown to generate extensive basaltic lava flows on all flanks of the shield volcano (e.g., Barberi and Varet 1970; Field et al. 2012; Pin et al. 2024).

On the southern end of the range, Hayli Gubbi forms a gently sloping edifice up to ca. 440 m asl with a NW–SE trending summit depression of ~ 0.7–1 km width. Along the walls of the summit depression, remnants of a lava lake have been described (Varet 2018). The edifice is cross-cut by NNW–SSE trending fractures and aligned spatter and cinder cones and pit craters (Acocella 2006). Some of these cones and fractures appear to have been the source of morphologically young lava flows, emanating both from the northern sector of the summit depression, and from the extensive fractures on the southern flank. The longest lava flow extends ~ 20 km from a row of fissure-fed cones located ~ 5 km south of the summit and is largely confined by a graben over a length of ~ 11 km, before it fans out into the sedimentary plain just north of Lake Afrera (Fig. 1b).

On the slopes of Hayli Gubbi near the crater rows, the southern lava flow seems partially covered by one or more smaller lava flows that are mostly restricted to the graben, with a few narrow lobes extending outward on the upper slopes. Most eruptive products at Hayli Gubbi are described as basaltic (picritic olivine basalt to plagioclase-phyric basalt) composition, including the southern flows (Varet 2018). In contrast, the lava flows emanating from the northern sector of the summit depression are reported as trachytic (Varet 2018) and we suspect these may correspond to the few published chemical data on Hayli Gubbi lava flows, which are of basaltic trachyandesite and trachyandesite composition (Barrat et al. 1998).

Prior to the November 2025 eruption, a prominent crater with ~ 250 m crater diameter existed centrally in the summit depression. Fumarolic activity has been repeatedly observed in this summit crater (Varet 2018), and pre-eruption satellite images indeed show its inner walls to be affected by hydrothermal alteration. The trachytic lava flows on the northern slopes emanate from an irregular dome-shaped feature just north of the summit crater.

According to Varet (2018), the youngest pahoehoe flow of Hayli Gubbi (presumably the southern one, though this is not specified) overlies sedimentary strata dated to 8200 years ago. However, the source cited for this statement (Roubet 1969) does not report interbedded lava flows and sedimentary strata in the proximity of Hayli Gubbi. Instead, the reported dates, mostly U-Th dates on corals and bivalves from sections west of Erta Ale and Dallol, are all mid-to-late Pleistocene, ranging from ~ 45 to ~ 230 ka (Roubet 1969). To the best of our knowledge, no absolute chronology exists on any prehistoric eruptions at Hayli Gubbi. Given the young geomorphological appearance of its craters and lava flows, and its structural connection with Erta Ale, it is likely that Hayli Gubbi has repeatedly erupted throughout the Holocene.

## Data and methods

We drew on several publicly available sources of geophysical and remote sensing data, combined with collection of a sample of new tephra. Continuous seismic waveform data from the three closest real-time and permanent stations in Ethiopia (FURI), Djibouti (ATD) and Yemen (DAMY; Fig. 1) were downloaded from EarthScope and manually inspected with the Seismic Analysis Code (SAC). We sourced earthquake locations from the National Earthquake Information Centre (NEIC) catalogue (USGS 2024) and the moment tensor solutions from the gCMT catalogue (Dziewonski et al. 1981; Ekström et al. 2012).

The surface deformation was measured by InSAR using data from the European Space Agency’s (ESA) Synthetic Aperture Radar (SAR) satellite Sentinel-1. Interferograms were processed using the InSAR Scientific Computing Environment (ISCE2) software package (Rosen et al. 2012). For the processing, we used a 1-arc-sec (∼30 m resolution) SRTM DEM (Farr et al. 2007) for co-registration of the SLCs topographic phase removal and interferogram geocoding. We also filtered the interferograms using a Goldstein adaptive power spectral filter with strength of 0.5 (Goldstein and Werner 1998) and unwrapped them using the ICU branch-cut algorithm. To model the deformation, we assumed tensile dislocation sources in an elastic half-space with a Poisson’s ratio of 0.25 and a shear modulus of 30 GPa. We inverted the interferograms for the source parameters using a nonlinear Monte Carlo simulated annealing inversion (Pagli et al. 2012) assuming uniform contraction of the sources. We also computed interferometric coherence images as these are a measure of the changes in the scattering properties of the surface and can be used as a proxy for mapping areas of ash deposition.

Infrared satellite observations were sourced from the Visible Infrared Imaging Radiometer Suite on the Suomi-NPP and JPSS-1 satellites, with a spatial resolution of 750 m (VIIRS; Campus et al. 2022) and reported by the MIROVA service (Coppola et al. 2020). We used Copernicus Sentinel-2 L2A optimised natural colour satellite images (ESA 2025) and MODIS images acquired by NASA’s Aqua satellite (NASA Earth Observatory 2025) for optical observations of the volcanic edifices and craters, visible degassing, the eruption plume and deposit traces. We also drew on eyewitness reports by locals. Key observations not covered by our datasets are cited from other sources such as COMET (2025a, 2025b, 2025c).

An ash sample was collected the day after the eruption on the western flank of Erta Ale Volcanic Range, ~ 11.5 km NW of the Hayli Gubbi summit crater (Fig. 1b) and represents ashfall from the northern plume. Its grain size was measured by laser diffraction, and its componentry by optical and scanning electron microscopy–energy dispersive spectrometry (SEM–EDS) at the Université libre de Bruxelles (ULB), Belgium. Bulk rock major element concentrations were also determined at ULB using a manually powdered sample, prepared as a solution by alkaline fusion and measured by inductively coupled plasma–optical emission spectrometry (ICP-OES). Bulk rock trace elements were measured at the University of Southampton, UK, by digesting the ash sample in HF/HNO_3_ and then analyzing the solution by triple quadrupole inductively coupled plasma mass spectrometry (QQQ-ICP-MS). Finally, major element analysis of the tephra glass was undertaken at the University of St Andrews, UK, using an Electron Probe Micro Analyzer (EPMA). Full details of the instrument setup and analytical protocols are provided in Online Resource 1; data including on secondary standards are presented in Online Resource 2.

## The Hayli Gubbi eruption of 23 November 2025

### Deformation prior to the intrusion, from July 2025

**Fig. 2:**
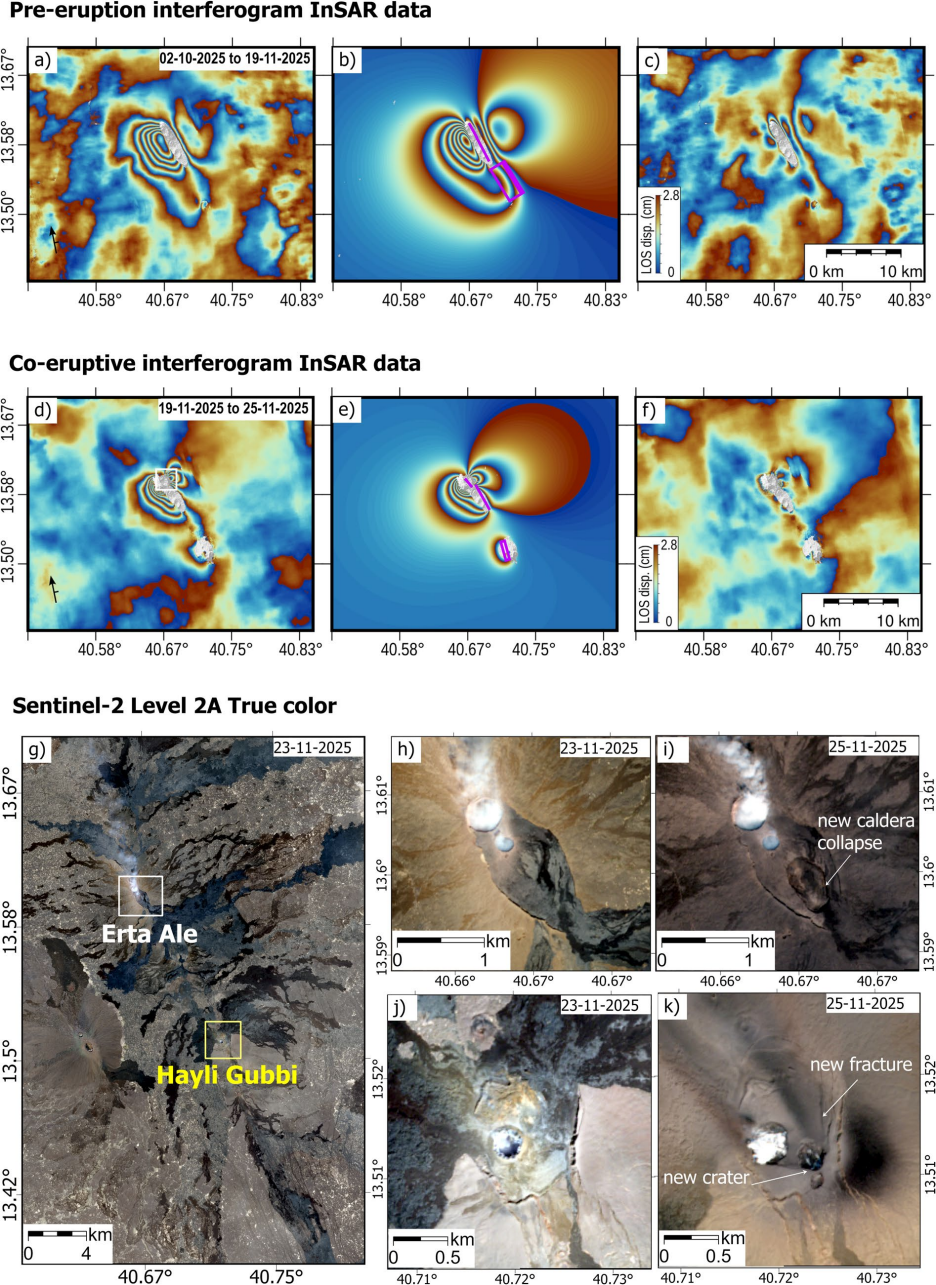
InSAR and optical data. (**a**) Sentinel-1 co-eruptive interferogram spanning 02-10-2025 to 19-11-2025; (**b**) model interferogram, assuming two dyke segments and a sill; (**c**) residual interferogram. (**d**-**f**) is the equivalent for the period covering the eruption, 19-11-2025 to 25-11-2025. The black arrow in (**a**) and (**d**) shows the satellite flight path with the black tick indicating the satellite Line-of-Sight (LOS). The magenta lines and rectangle in (**b**) and (**e**) represent the modelled dyke and sill projection at the surface, respectively. Positive values of LOS displacement indicate an increase in the satellite-to-ground distance. (**g**-**k**) Sentinel-2 surface reflectance (Level 2) in true colours. (**g**) overview; Erta Ale before (**h**) and after (**i**) the eruption; Hayli Gubbi before (**j**) and after (**k**) the eruption. The white rectangle in (**d**) and (**g**) marks the area shown in (**h**) and (**i**) and the yellow rectangle in (**g**) the area shown in (**j**) and (**k**). Dates are in DD-MM-YYYY format

Erta Ale had a small explosive eruption and collapse at its northern summit caldera on 15 July 2025, producing volcanic ash and sulphur dioxide plumes. This unusual explosive eruption was followed on 15–16 July 2025 by effusive activity emanating from the southern summit caldera, and continued activity at the northern caldera pit craters (COMET 2025a; La Rosa et al. 2025). Prior to this July 2025 eruption at Erta Ale, deformation patterns were interpreted as the result of a N-S striking dyke under Erta Ale’s northern caldera since January 2020, and a deflating sill between the southern caldera and Hayli Gubbi since at least January 2018 (La Rosa et al. 2025). A detailed analysis of the InSAR deformation patterns covering the 15 July 2025 eruption and the weeks after shows that this eruption was associated with a dyke intrusion that originated from Erta Ale’s northern summit caldera and propagated during a period of 25 days over 30 km southward beneath the axis of the ridge reaching the Afrera Plain. The dyke intercepted two shallow magma reservoirs at ~ 1 km depth, at the Erta Ale southern caldera and at Hayli Gubbi, where inflation occurred between 22 July and the end of the intrusion on 09 August 2025. The InSAR deformation patterns were modelled as a series of opening and contracting dyke segments as well as two inflating magma reservoirs at the Erta Ale southern caldera and Hayli Gubbi. The dyke intrusion opened to a width of up to 4 m and had an estimated total volume of ~ 0.4 km^3^ (La Rosa et al. 2025). InSAR shows that renewed ground deformation began at Erta Ale in late September 2025 (Fig. 2a–c) and continued until the Hayli Gubbi eruption on 23 November 2025 (Fig. 2d–f). The InSAR deformation is consistent with a contracting source beneath the Erta Ale caldera extending to Hayli Gubbi, where some minor uplift is observed. We modelled the deformation pattern with three contracting dyke segments reaching a total of 11 km in length, from the Erta Ale calderas to Hayli Gubbi. The two northernmost dyke segments are adjacent, vertical and extend from ~ 1 to 2 km depth beneath the Erta Ale calderas, while the southern dyke segment connecting to Hayli Gubbi dips 50°SW and is 2- to 3.5-km deep. The total volume of contraction of the dykes is 9.5 × 10^–3^ km^3^. No deformation source at Hayli Gubbi was included in the modelling as uplift is minor.

**Fig. 3:**
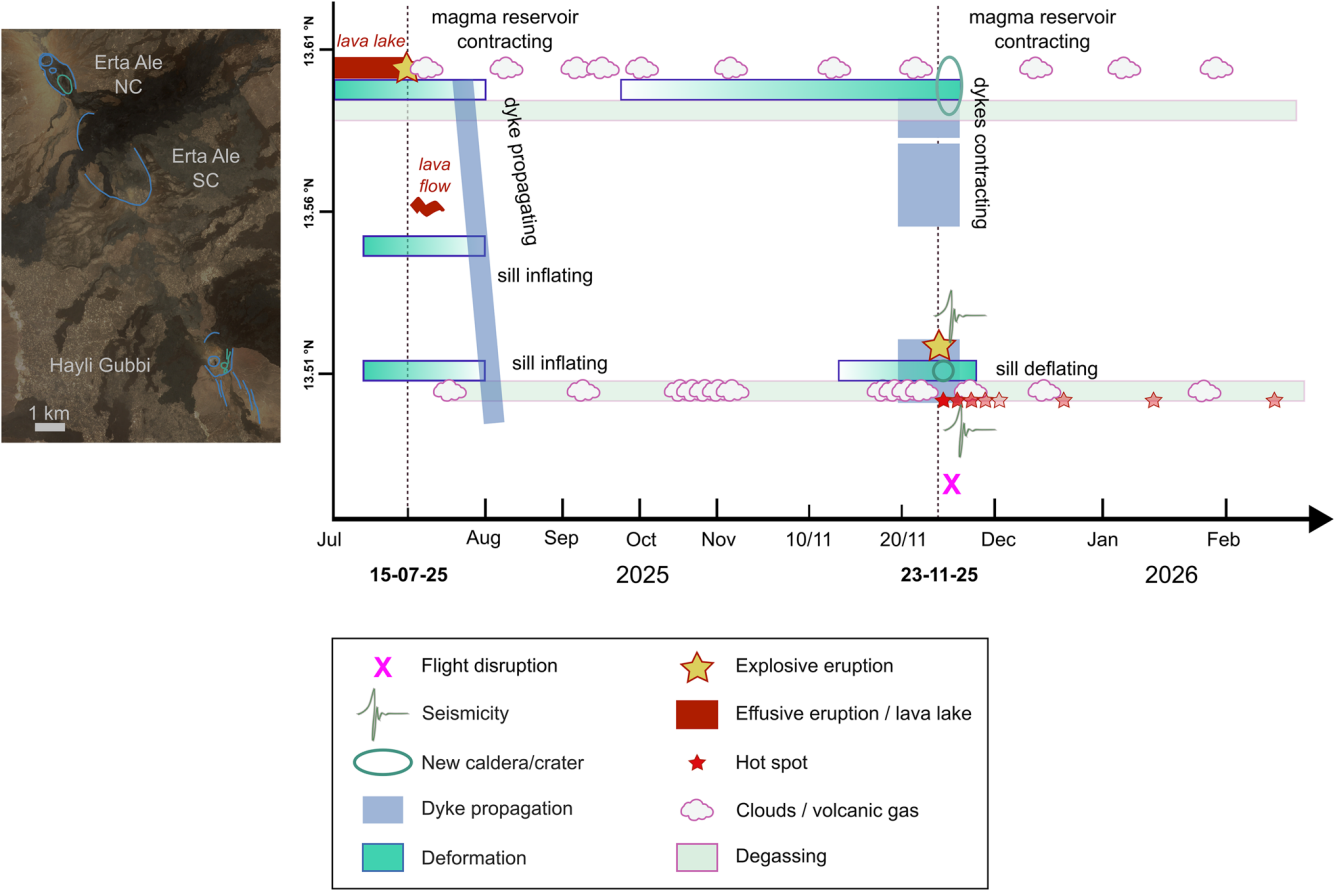
Summary timeline of activity between mid-July 2025, starting with an explosive eruption at Erta Ale’s northern caldera, and prior to and in the weeks and months following the 23 November 2025 Hayli Gubbi eruption. For more details on deformation signals and interpreted magma propagation prior to and during the July 2025 eruption, see La Rosa et al. (2025). Previously existing and newly crater and caldera features at Erta Ale and Hayli Gubbi indicated on Copernicus Sentinel-2 L2A-optimized natural-colour satellite image from 29-05−2025

A schematic overview of events since July 2025 is presented in Fig. 3. As early as 26 July 2025, Sentinel-2 satellite imagery shows the occasional appearance of diffuse white clouds in the central summit crater of Hayli Gubbi (COMET 2025b). These become especially dense in the period of mid-to-late October (or perhaps earlier, but no clear observations exist between 29-09−25 and 14-10−25) and stay confined to the central crater. The dense clouds in the crater are no longer visible from 08–11−25 onward, but reappear 10 days later, in the week preceding the eruption on 23 November. The appearance of these dense crater-confined clouds at Hayli Gubbi (as opposed to near-continuous visible degassing observed at Erta Ale’s northern caldera pit craters) may be the result of steam release from a perturbed hydrothermal system after (repeated) dyke intrusion. The first optical images of an explosive eruption generating a grey-brown ash plume were taken on 23 November 2025 08:31 UTC. Other images acquired 35 min earlier do not show the eruption (COMET 2025c) and so we can confidently constrain the eruption onset between 07:54 and 08:31 UTC.

### Geophysical signals associated with the 23 November 2025 eruption

**Fig. 4:**
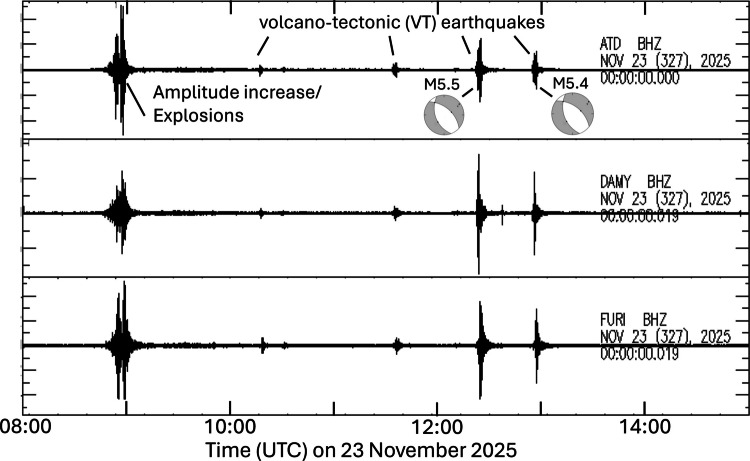
Vertical component seismograms of three regional broadband seismic stations (FURI: 8.8952°N–38.6798°E, ATD: 11.5307°N–42.8466°E and DAMY: 14.5613°N–44.3869°E) showing tremor between 08:44 and 09:10 UTC and four distinct volcano-tectonic earthquakes in the hours following the eruption. Moment tensors are from the global CMT program (Dziewonski et al. 1981; Ekström et al. 2012). ATD is part of the GEOSCOPE network (Institut de physique du globe de Paris (IPGP) and Ecole et Observatoire des Sciences de la Terre de Strasbourg (EOST) 1982)

The seismic activity associated with the eruption was recorded by permanent seismic stations in Ethiopia (FURI), Djibouti (ATD) and Yemen (DAMY) (Fig. 4). The primary signals were of continuous low frequency and high amplitude during 08:44–09:10 UTC. The seismic signal increased in amplitude to two distinct peaks in energy within 08:50 and 09:00, interpreted to be two distinct volcanic explosions (Limberger et al. 2026). The fact that the signal is only clearly visible from 08:44 potentially means the onset of the eruption at 08:31 (or in the minutes before) was initially not energetic. Indeed, Limberger et al. (2026) suggest the occurrence of low-amplitude pulse-like precursors of relatively high frequency starting at 08:30 UTC, before the main eruptive phase, though they do not relate them to an eruptive process, which was clearly already ongoing.

The main signal was still followed by at least four discrete earthquakes during the following four hours. The two largest amplitude earthquakes of these with Mw 5.5 and 5.4 were located by the NEIC catalogue to be 2 km SSE of Erta Ale (Fig. 1b), though the large error bars of ~ ± 15 km mean that they could feasibly be from Hayli Gubbi, or anywhere between the two volcanoes. The two earthquakes have global CMT moment tensor solutions consistent with normal faulting, with nodal planes oriented NNW-SSE, parallel to the rift strike (Fig. 1b). The overall seismic activity observed associated with this eruption is low compared to the intense and protracted seismicity observed during the Fentale-Dofen seismic crisis in 2024–2025 (Keir et al. 2025; Lewi et al. 2025). This may indicate the shallowness of the dyke and volcano dynamics at Hayli Gubbi or be due to limited pre-eruptive tectonic stress.

The surface deformation associated with the eruption was measured by InSAR. The shortest co-eruptive interferograms spanning 19–25 November 2025, show a deformation pattern consistent with contraction of a dyke-shaped reservoir under the Erta Ale summit calderas and extending to the Hayli Gubbi volcano where concentric contraction is also observed (Fig. 2d). The interferogram was modelled assuming three different contracting sources. Two vertically oriented planes resembling contracting dykes (Okada tensile dislocation) are located under the Erta Ale calderas and extending towards Hayli Gubbi. A third horizontal plane resembling a contracting sill is under Hayli Gubbi volcano (Fig. 2e). The northern dyke segment is 1.5 km long, extending from the surface to 0.9 km depth with a contraction of 1.2 m. The southern dyke segment is 3.9 km long, extending from 0.1 km depth to 2.1 km depth with a contraction of 0.25 m. The contraction of the two dyke segments corresponds to a total volume decrease of 3.5 × 10^–3^ km^3^. The contracting sill at 1.5 km depth under Hayli Gubbi is 2.5 km long and 0.5 km wide with a contraction of 0.3 m, corresponding to a volume decrease of 0.4 × 10^–3^ km^3^. This model explains the majority of the InSAR displacement, but a small residual signal remains midway between the southern tip of the dyke and the sill (Fig. 2f) suggesting a physical connection between Erta Ale and Hayli Gubbi not explained by our modelling. The observations are consistent with visible evidence from optical satellite images for collapse events in the southern sector of the northern Erta Ale summit caldera and at Hayli Gubbi at some point between 23 and 25 November at 07:55 UTC (Fig. 2g-–k). The collapse at Erta Ale generated a new caldera of ~ 650-m long (NW–SE) by ~ 350-m wide and at Hayli Gubbi the existing main crater enlarged from 200 to 350 m in diameter and a new crater formed, SE of the main one, with a 200-m diameter. In early February 2026, no visible lava lake was present in the craters of Erta Ale’s northern caldera.

### Local reports

Informal discussions with three local inhabitants the day after the eruption revealed they noted “smoke” coming from the crater during the three days prior to the eruption. This is consistent with the observation of dense white clouds on optical satellite imagery (COMET 2025c). In addition, they experienced ground shaking (felt moderate-magnitude earthquakes) in the three to four days prior to the eruption and reported the opening of fissures surrounding the crater. They also reported unusual sounds and noise prior to and during the eruption. Finally, during the eruption, there was limited visibility across the surrounding area because of ash in the atmosphere.

### Eruptive phenomena

**Fig. 5:**
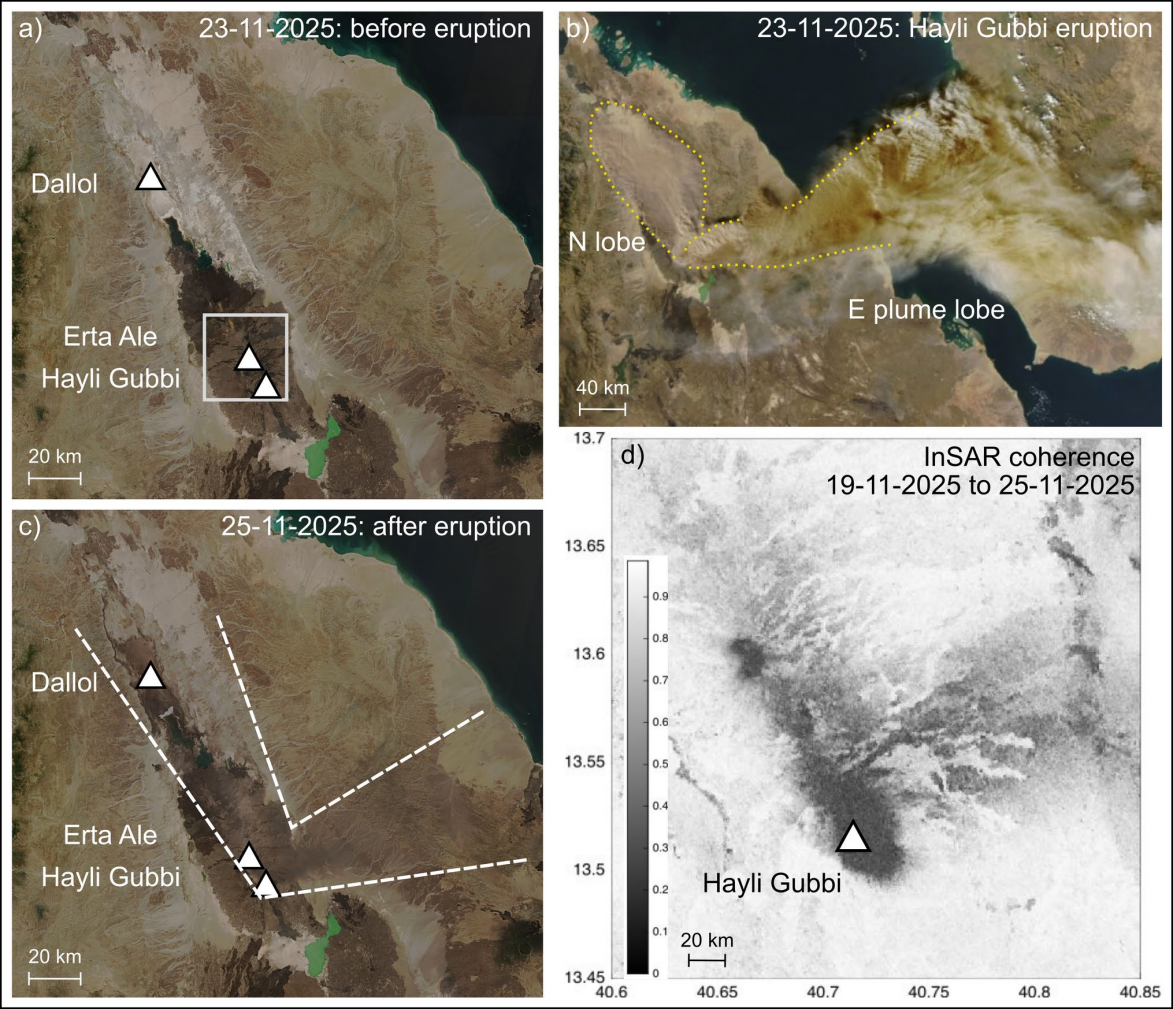
**a**, **c** Copernicus Sentinel-2 L2A optimized natural colour satellite images of the Danakil Depression (**a**) before and **c** after the Hayli Gubbi eruption. **b** MODIS image acquired by NASA’s Aqua satellite approximately four hours after the eruption was first detected, showing a prominent grey-to-yellow-coloured ash plume spreading east towards the Arabian Peninsula, and a secondary grey-to-cream-coloured plume at lower elevation spreading north towards Dallol (NASA Earth Observatory 2025). The near-elliptical dispersal of the northern lobe is indicated by a yellow dashed line. **c** The spatial extent of the new dark ash cover, especially visible where it coats light-covered sedimentary rocks and basin floor cover, including in the area around Dallol. **d** InSAR coherence map between radar images acquired before (19 November 2025) and after (25 November 2025) the eruption. Values in the coherence map range from 0 for no coherence (surface characteristics have completely changed between acquisitions) to 1 for high coherence (surface characteristics have not changed between acquisitions). Regions of low coherence are dark and primarily show the spatial extent of new tephra deposit

Photos of the eruption show that it formed a sustained plume with two distinct lobes: a lower-elevation lobe with a grey-cream colour spreading to the northwest at ~ 4–5 km elevation and a ~ 10–12 km high plume with lighter colour dispersed towards the east (Fig. 5b). The height of the plume lobes is estimated by the NOAA JPSS Visible Infrared Imaging Radiometer Suite (Bachmeier 2025). The eruption plume was observed from a far distance, for example, from the towns of Logia and Semera at almost 200 km south of the volcano (Fig. 1a; Afar Communication Bureau 2025). Some photos posted by the Afar Communication Bureau (2025) show ash-rich plumes with a wide base and which may represent ash elutriation from localized density currents, though the exact locations of where the photos were taken from cannot be confirmed.

**Fig. 6:**
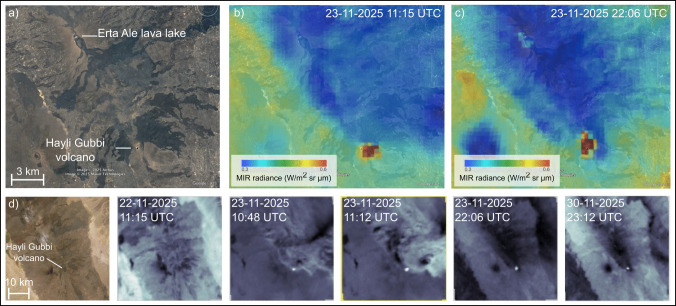
Thermal anomalies detected by the Visible Infrared Imaging Radiometer Suite (VIIRS, Middle InfraRed (MIR) radiation at ~ 750 m resolution; Campus et al. 2022) and reported by MIROVA (Coppola et al. 2020). **a** Visible spectrum Google Earth image showing the spatial extent of (**b**) and (**c**), which show the thermal anomaly at Hayli Gubbi detected on 23 November 2025 in red (scale bar of MIR radiance from 0.3 to 0.6 W m^−2^ sr^−1^ µm^−1^). **d** The lower panels show a more complete time series of the thermal anomaly with the hotspot shown in white; each panel shows the same spatial extent. The two middle panels at 10:48 and 11:12 UTC also show the ash plume

Infrared satellite images (Coppola et al. 2020; Campus et al. 2022) first report a localized hotspot ~ 2 km in diameter at Hayli Gubbi on the first available image of 23 November at 10:48 UTC, just over two hours after the start of the eruption (Fig. 6). The hotspot remains in the same position and progressively decreases in intensity for just over a week (Fig. 6d). In the months following the eruption, a faint hotspot reappears occasionally in the same location, most recently (at the time of writing) on 16 May 2026. Because no molten lava was observed immediately after the eruption, the hotspot may instead represent hot fumarolic gases issued from the central crater.

Optical imagery from the Copernicus Sentinel-2 satellites of the summit depression of Hayli Gubbi before and after the eruption shows the appearance of two new circular craters of ~ 230 m and ~ 100 m diameter to the SE of the pre-existing central crater, together with new visible fractures which are oriented NNE-SSW and WNW-ESE (Fig. 2j and k). Some minor fumarolic activity seems present in these craters until at least 8 December 2025, though less vigorous than in the central one. We suspect it is therefore likely that the thermal signal is also located on the central pre-existing crater. Throughout December 2025 and January–February 2026, dense white clouds and increasingly noticeable patches of yellow deposits are visible in and around this central crater (Fig. 3). A field visit in early February 2026 by one of us (AZT) indeed confirmed ongoing vigorous degassing with a strong sulphur smell from the central crater, and fresh sulphur deposits in its vicinity. During this posteruptive period, rumbling noises are still heard near the crater.

The appearance of the second crater (and a third minor one) at Hayli Gubbi and two distinct plume lobes with different colours, dispersing at different elevations, raises the question whether the plume lobes issued from the same crater and/or at the same time. It is also noticeable that the initial eruption onset (i.e. visual observation of an ascending ash-laden plume) occurred at least 13 min before the first clear seismic signal, and that seismicity became progressively more intense. It is therefore possible that the first plume ascended during the near-aseismic phase, and the second one was associated with the higher-amplitude signal including two distinct explosions (Fig. 4), potentially associated with a vent-clearing event of the second and third crater. Unfortunately, the satellite imagery does not allow us to unequivocally distinguish the vent source of the two ascending plumes, nor allow us to reconstruct a clear time series of plume ascent and relate it to the recorded seismicity.

### Eruption deposit

**Fig. 7:**
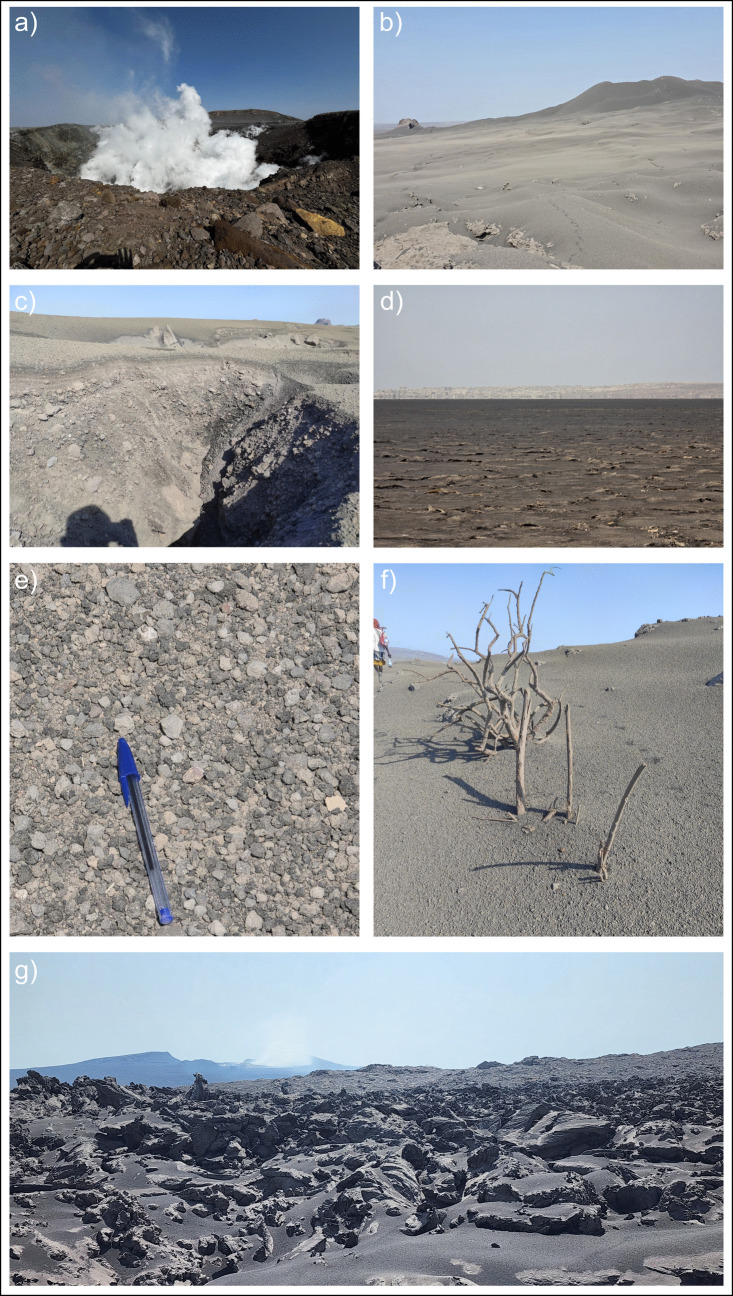
Photo panel of Hayli Gubbi 23 November 2025 deposits taken on 25-11-25 (**a**), (**b**), (**d**), (**e**), and on 01-02-26 (**c**), (**f**), and (**g**). (**a**) steaming central crater with dm-scale hydrothermally altered lithic blocks scattered near the NW rim; (**b**) pre-existing irregular lava flow surface covered with fresh dark grey volcanic ash, photo taken within 1 km of the NW rim, looking NNW; (**c**) section through a proximal deposit filling in topography of preexisting lava flow surface(13.5170°N – 40.7137°E); (**d**) distal ash covering Dallol salt plain at ~90 km NNW of the source; (**e**) lapilli-sized particles deposited at less than 1 km to the NW of the central crater (**c**); (**f**) ash fall covering vegetation, ~1.3 km NW of the main crater; (**g**) ash fall covering pre-existing lava flow surface, at ~2.4 km NW (looking SE) of the degassing Hayli Gubbi crater observed in the background

We visited the accessible NW flank of the volcano in the days after the eruption (DA, DK, HG, and OA) and in early February 2026 (AZT). The Eastern flank with the two new craters is more difficult to access and so most of our observations from the ash fallout on the ground likely represent the northern lobe of the plume (Fig. 5b), and proximal near-vent fallout around the central crater. Our observations show that the eruption deposited bombs and blocks up to ~ 1-m large of country rock along with dark, fresh vesicular lapilli near to the vent/crater (Fig. 7a and b). The largest fragments found within a range of ~ 500 m around the crater are usually hydrothermally altered and commonly form impact sags on the ground.

In the proximal location near the vent (within less than 1 km distance), we observe a relatively well-sorted layer of ~ 15 cm, likely emplaced as fallout (Fig. 7e), overlying in some places ~ 75 cm of poorly sorted deposit of the same colour (Fig. 7c). This lower part of the deposit was likely emplaced by a localized pyroclastic density current infilling the preexisting topography made of the prehistoric lava flows.

The fresh deposit has a dark brown grey to black colour, and the grain size and thickness of the northern fall deposit decrease away from the crater, in the NW direction. At ~ 2 km distance to the NW, we find a maximum grain size of ~ 3.5 cm and a thickness of ~ 5–10 cm; at ~ 11.5 km distance the thickness reduces to 5 mm. In the latter location, we took a sample of the deposit for grain size and chemical analysis. The grain size shows a median of ~ 10.6 µm and 10% and 90% percentile of 1.1 and 100 µm, respectively.

The volcanic ash covers the ground surface, vegetation (Fig. 7f), water bodies and locally some houses. Ash fall was observed as far as the Dallol salt plain (~ 90 km from the vent), which turned dark brown to black as a result (Fig. 7 d). A comparison of satellite imagery taken the day before and after the eruption, also shows the appearance of a light to dark brown veil deposited across Afar, primarily in the NNW direction of Dallol and to the ENE (Fig. 5a and c).

Assuming a distal thickness at Dallol of ~ 1 mm and a strongly elliptical dispersal pattern following the observed northern plume lobe and its visible ash deposit (Fig. 5c and d), we identify a two-segment exponential decay of thickness versus distance from the central crater. Using the method of Fierstein and Nathenson (1992), we estimate a minimum deposit volume of order of ~ 1.6 × 10^7^ m^3^ for the northern lobe. Although we do not have thickness and volume constraints on the eastern lobe, we estimate it to have been of at least 1–2 times the size of the northern lobe, because its plume ascended at higher elevation and satellite observations suggested higher ash concentrations in the atmosphere (Toulouse VAAC 2025). This would give a conservative estimate of the total deposit volume of ~ 3–4.5 × 10^7^ m^3^, corresponding to a Volcanic Explosivity Index (VEI) (Newhall and Self 1982) of the entire eruption around 3, consistent with the observed plume heights. Future detailed mapping of the deposit may allow refining these volume estimates.

### Chemical composition

An ash sample was taken the day after the eruption on the W flank of Erta Ale Volcanic Range, at ~ 11.5 km NW of the Hayli Gubbi summit crater (Fig. 1b) and thus represents ashfall from the northern plume. The sieved sample shows a limited proportion of ~ 20–25% of visibly vesicular black, and thus presumably juvenile, particles. Other components include free crystals of both feldspar and ferromagnesian minerals, dense red-orange hydrothermally altered particles and dense dark brown to black lithic particles. Backscatter images and qualitative EDS profiles confirm the presence of moderately vesicular clasts with a glassy to—in most cases—microlite-rich groundmass (Online Resource 3: Fig. 1). The crystal cargo of the vesicular—and thus presumably juvenile—clasts is composed of plagioclase, clinopyroxene and Fe-Ti oxides. We also find free crystals of plagioclase and blocky microcrystalline clasts.

**Fig. 8:**
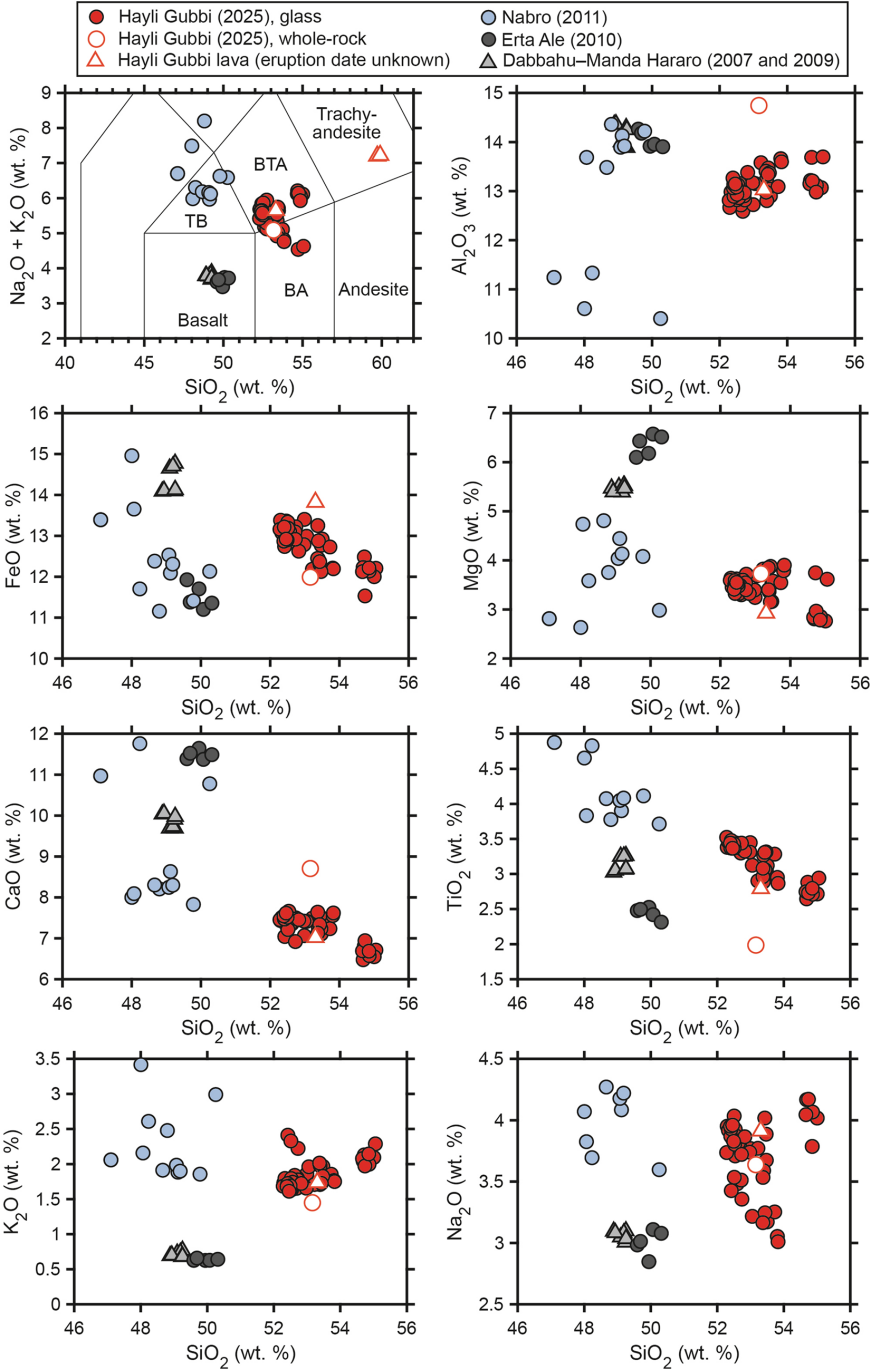
Major element geochemistry of the 2025 Hayli Gubbi tephra. In the total-alkali versus silica diagram (top left panel) the abbreviations are TB: trachybasalt, BTA: basaltic-trachyandesite, and BA: basaltic-andesite. For Hayli Gubbi, red filled circles show glass analyses while the white filled circle shows whole-rock analysis. For comparison we plot Hayli Gubbi lava analyses from Barrat et al. (1998) (two trachyandesites and one basaltic trachyandesite), and from recent eruptions from Erta Ale (2010, glass analyses from Field et al. 2012), Dabbahu MHRS (2007 and 2009, whole-rock analyses from Ferguson et al. 2010) and Nabro (2011, glass analyses Donovan et al. 2018)

The Hayli Gubbi tephra show a relatively tight SiO_2_ range (52–55 wt. %). When plotted on a total alkali versus silica (TAS) diagram it straddles the basaltic andesite–basaltic trachyandesite boundary (with more analyses in the latter; Fig. 8). The glass shows a main compositional cluster between 52 and 54 wt. % SiO_2_. An additional compositional grouping shows > 54 wt. % SiO_2_ and generally lower CaO, TiO_2_ and higher K_2_O and Na_2_O when compared to the majority of glass analyses (with 52–54 wt. % SiO_2_). These differences (i.e., the increasing SiO_2_, decreasing CaO and TiO_2_ and increasing alkalis) would be consistent with the expected fractionating assemblage (i.e., plagioclase feldspar, pyroxene and Fe–Ti oxides). Thus, the range in chemistries likely reflects variable fractionation of the melt. Given the limited number of geochemical analyses, it is not yet possible to determine whether the clustered compositions reflect a lack of samples for a melt that has undergone variable crystal fractionation, or if different magma batches with diverse magma chemistries were involved in this eruption. Comparing the whole-rock chemistry of the Hayli Gubbi tephra to the glass analyses shows good correspondence on SiO_2_, MgO and Na_2_O but notable offsets on other oxides (Fig. 8). Given the abundance of hydrothermally altered and other lithic material in the ash, it is clear that the whole-rock composition is not entirely representative of the juvenile magma.

In comparison with recent eruptions from Afar, i.e., Erta Ale (2010, Field et al. 2012) and Dabbahu MHRS (2007 and 2009, Ferguson et al. 2010), the Hayli Gubbi samples are more chemically evolved. They are also more SiO_2_-rich than the tephra from Nabro (2011, Donovan et al. 2018), although with comparable alkali contents. A handful of previous whole-rock analyses of Hayli Gubbi lavas samples from Barrat et al. (1998) show trachyandesite and basaltic trachyandesite compositions. Despite the difference in methods (whole rock vs. EPMA) and eruptive styles (lava vs. tephra), the geochemistry of the 2025 eruptive products bears a remarkable geochemical affinity to this past effusive eruption activity of Hayli Gubbi.

### Eruption impacts

Whilst the area is overall sparsely populated, some nearby settlements experienced minor ashfall. Because the eruption happened in a remote area, direct impact in terms of casualties or damage to property or infrastructure was not reported. Only five camels and fifteen goats disappeared according to the three residents we spoke to. However, the widespread volcanic ash cover to the northwest led to temporary interruption of salt extraction activities in the Dallol salt plain north of the Erta Ale Volcanic Range, and near Lake Afrera to the south, during 1 to 2 days. To the best of our knowledge, the eruption did not disrupt access to visit the region.

Ash dispersal to the East moderately disrupted air traffic in South Asia, mostly in India, causing delayed and some cancelled flights on 24 and 25 November (Labanauskaite 2025). The Toulouse Volcanic Ash Advisory Centre (VAAC) issued an advisory notice on 23 November 2025 at 14:55 UTC and at 16:22 UTC, indicating an ongoing explosive eruption in Afar (Toulouse VAAC 2025). The next advisory at 20:00 UTC indicated that the eruption had stopped; however, the following day modelled concentration maps of low, medium and high airborne ash concentration showed ash (and SO_2_) dispersal over South Asia towards India. On 24 November 2025 at 17:00 UTC the Toulouse VAAC indicated that the ash plume detected over Northern India was spreading quickly towards China. The responsibility for the ash event was transferred from Toulouse to the Tokyo VAAC at 21:30 UTC. At this stage, the aviation colour code was nil, and on 26 November 2025 at 05:57 UTC the most recent advisory released stated that volcanic ash dissipated on satellite imagery (Tokyo VAAC 2025).

## Interpretation and perspectives

### Eruptive sequence

InSAR observations reveal that in July 2025 until early August a ~ 30 km long dyke was intruded under the Erta Ale ridge, and it was fed by a dyke-like contracting reservoir under the Erta Ale summit calderas (La Rosa et al. 2025). This dyke-like reservoir geometry was also observed during previous intrusions of shorter dykes (~3-km-long) in 2008–2010 and 2017–2019 (Moore et al. 2019; Xu et al. 2020), suggesting this is the long-term reservoir geometry of the Erta Ale magma plumbing system. During the propagation, the July dyke intersected two reservoirs at 1-km depth under the southern Erta Ale caldera and at Hayli Gubbi, where inflation occurred. This suggests that lateral dyking might be a mechanism for feeding other shallow on-rift magma chambers of the Erta Ale Volcanic Range. In the months leading to the Hayli Gubbi explosive eruption (September–November 2025), renewed contraction of the dyke-like reservoir under Erta Ale extended to Hayli Gubbi where there was some minor inflation. The dyke volume contraction is not balanced by inflation at Hayli Gubbi but this could be explained by magma compressibility effects in shallow reservoirs with different geometries (Rivalta and Segall 2008). Finally, the co-eruptive InSAR model (19–25 November) indicates that contraction of the dyke-like reservoir under Erta Ale extended to Hayli Gubbi and was simultaneous to the deflation of a 1-km depth sill at Hayli Gubbi. We interpret this as progressive magma transfer from Erta Ale to Hayli Gubbi from September 2025 that culminated in the 23 November explosive eruption, enabled by a connection established between the magmatic systems during the earlier July dyke intrusion.

Satellite data suggests the eruption started at 08:30 UTC. There were two main eruption phases, one causing a low-elevation plume to the north, and one causing a higher elevation plume to the east. We interpret the lack of seismic energy during the first ~ 15 min of the eruption to indicate that the low-elevation plume formed in the first eruption phase. In contrast, we interpret the delayed onset of the observed energetic seismic signal at 08:44–09:10 to be likely associated with the eruption forming the higher elevation plume, which therefore likely was the second eruption phase. The timing and multiple pulses of energy suggest this second phase was protracted for 25 min and involved several pulses.

The glass chemical composition of the analysed Hayli Gubbi tephra is largely consistent with fractional crystallisation of plagioclase, clinopyroxene and Fe-Ti oxides, however, shows varying SiO_2_ and other major element oxide contents. These variations may be due to the presence of different magma batches. Considering the dyke propagation from Erta Ale to Hayli Gubbi and preexisting shallow reservoirs under Hayli Gubbi, it is indeed possible that multiple magma bodies interacted before eruption. This could also explain the occurrence of two distinct plumes. However, further and more detailed coupled geophysical, volcanological and geochemical analyses are required to disentangle such processes and better understand the Erta Ale–Hayli Gubbi plumbing systems and eruption dynamics and associated hazards.

### Volcanic hazards and monitoring

About 50–55 potentially active Holocene volcanoes are known in the Main Ethiopian Rift and Afar Depression combined (the latter including volcanoes in Eritrea and Djibouti). All historically documented eruptions in Ethiopia occurred in Afar, mostly at Erta Ale (Global Volcanism Program 2025). Some have also occurred in Djibouti and Eritrea, most notably at the Dubbi and Nabro volcanoes (e.g., Wiart and Oppenheimer 2000; Goitom et al. 2015). Other recent eruptive activity in Ethiopia occurred at the Dabbahu–Manda Hararo segment, where repeated dyking in 2005, 2007 and 2008 resulted in a few eruptive events producing lava flows and very small-scale explosive activity (e.g., Ferguson et al. 2010; Yirgu et al. 2014).

In November 2008, a moderately explosive VEI 3 eruption with estimated plume height of ~13–15 km, and associated lava flow, occurred at Alu-Dalafilla, on the northwestern tip of the Erta Ale Volcanic Range (Global Volcanism Program 2008). Physical descriptions of this eruptive event are scarce; however, just like the 2025 Hayli Gubbi eruption, it also seems to have been preceded by a lateral dyke propagation from Erta Ale towards the north into the Alu-Dalafilla plumbing system (Pagli et al. 2012). The chemical composition of the lavas is assumed to be basaltic (Watts et al. 2020) though direct analyses are lacking. More detailed investigations of the past volcanic history, magmatic plumbing systems across the Erta Ale Volcanic Range and their structural and geochemical interactions are required to better understand the potential eruptive activity at any of the volcanic centres of the range. Such fundamental (geological) data would support high-quality data-supported probabilistic volcanic hazard assessments (e.g. Tierz et al. 2020).

Whilst developments in satellite-based monitoring of ground deformation have revolutionized volcano monitoring in the last few decades, and will continue doing so in the future, including (or especially) in the absence of ground-based networks (e.g., Biggs and Wright 2020; Poland and Zebker 2022), regular high-resolution monitoring of seismic unrest and short-term forecasting will still require a minimum of on-the-ground instruments. Particularly the absence of a dedicated permanent seismic network in Afar (and elsewhere in Ethiopia) complicates the regular monitoring of seismic activity at high resolution and thus the potential for forecasting eruptive events (e.g., Lewi et al. 2025). International collaborations are highly valuable, but are usually focused on scientific interpretations and may present a risk of overshadowing the contributions and expertise of locally based scientists (IAVCEI-INVOLC 2024). A dedicated volcano observatory with clearly defined collaboration policies and communication strategies would represent the crucial link between volcano monitoring and decision-makers during a volcanic crisis (Lowenstern et al. 2022). Such an institution could also be the central avenue of communication to local communities and stakeholders, both prior to and during volcanic crises, and perform a crucial role in raising awareness about volcanic and other natural hazards.

Although the 23 November 2025 eruption was short-lived, the volcano remains restless (Fig. 3). Whilst impact was limited because of the remote location, it was not entirely nihil, and future events could well cause more disruptions, e.g. from lava flows or ash fall reaching the inhabited Afrera plain. Particularly the distal dispersal of ash on a regional or even global scale may severely disrupt aviation and supply chains, resulting in important financial impacts at a regional or even global scale. Continued intense degassing and ground deformation at Hayli Gubbi (and Erta Ale) call for close follow-up and monitoring of gas flux and composition, seismicity and deformation. Such monitoring will reinforce the capacity to forecast any future activity but also improve the understanding of the dynamics of the Erta Ale Volcanic Range and other similar systems across Afar.

## Conclusions

The 23 November 2025 Hayli Gubbi eruption demonstrates the potential for moderate-size explosive eruptions in Afar, the most geodynamically active region in Ethiopia. Dyke propagation from Erta Ale to Hayli Gubbi occurred already several months before, and sill inflation renewed in the days before the eruption. The VEI 3 eruption produced two ash plumes dispersing at different elevation and leaving a thin ash cover across Afar. Ash from the northern plume has a basaltic (trachy)andesitic composition and is heavily enriched by hydrothermally altered and other lithic particles. Intense degassing continues several months after the eruption. We anticipate that future detailed studies on the geophysical unrest, magmatic degassing, petrology and physical volcanology of the eruption will be able to increase our knowledge of the basaltic-trachytic volcanic system of Hayli Gubbi and its connection to the Erta Ale Volcanic Range.

## Supplementary Information

Below is the link to the electronic supplementary material.

ESM 1 (PDF 45 KB)

ESM 2 (XLSX.48.5 KB)

(PNG 994 KB)

ESM 3 (TIF.11.0 MB)

## Data Availability

The NEIC catalogue (USGS 2024) is available at https://earthquake.usgs.gov/earthquakes/search/. Seismic data from ATD, FURI and DAMY are available from Earthscope https://service.iris.edu/irisws/timeseries/docs/1/builder/. ATD is in the GEOSCOPE(G) network (10.18715/GEOSCOPE.G) (IPGP and EOST, 1982). Global CMT catalogue is available at https://www.globalcmt.org/CMTsearch.html. Sentinel-1 Single Look Complex (SLC) and Sentinel-2 acquisitions are from the European Space Agency (ESA) and Copernicus programme (https://browser.dataspace.copernicus.eu; ESA, 2025). Files accessed through Alaska Satellite Facility (ASF) Data Search Vertex https://search.asf.alaska.edu/#/.

## Code availability

Seismic data plotted in SAC, available from Earthscope at https://ds.iris.edu/files/sac-manual/. Interferograms were processed using InSAR Scientific Computing Environment (ISCE) v2 (Rosen et al. 2012), which is open source from NASA‐JPL at https://github.com/isce-framework/isce2.
