# Supplementary material for: The first documented volcanic eruption of Hayli Gubbi, Afar, Ethiopia
Source: Bull Volcanol. 2026 Jun 30;88(7):78. doi: 10.1007/s00445-026-01997-3 (PMC13319164; doi:10.1007/s00445-026-01997-3)
Supplement: Supplementary file 1 — (PDF 45 KB) [file 445_2026_1997_MOESM1_ESM.pdf]

Online Resource 1

The first documented volcanic eruption of Hayli Gubbi, Afar, Ethiopia

Dereje Ayalew · Karen Fontijn · Faysel Sefa Abdu · Derek Keir · Amdemichael Z. Tadesse · Carolina Pagli · Alessandro La Rosa · Pablo Tierz · Abate Melaku · William Hutchison · Patrick Sugden · Gezahegn Yirgu · Asfie M. Nigussie · Osman Ahmed · Hindeya Gebru · Martin F. Mangler · Matthew J. Cooper · Atalay Ayele

Methods description (componentry and geochemistry)

The sample grain size was measured using a Malvern Panalytical Mastersizer 3000 at the Laboratoire de Glaciologie of the Université libre de Bruxelles (ULB), Belgium. The sample was wet sieved at 25 µm and set in EpoFix resin and prepared as a polished section. Componentry was qualitatively inspected by optical microscopy and Scanning Electron Microscopy - Energy Dispersive Spectrometry using an SEC SNE-4500M Plus B fitted with a Bruker Quantax EDS detector, at the Laboratoire G-Time of ULB.

We undertook glass major element geochemical analysis of the ash sample at the University of St Andrews. The sample was sieved to 150-250 µm size fractions and mounted in epoxy resin (EpoThin2). Surfaces were then ground down and polished using diamond paste (9, 6, 3, 1 µm), with a final polish using 0.3 µm aluminium oxide slurry. Glass major element concentrations were determined using a JEOL iSP100 Electron Probe Micro Analyser with 5 wavelength-dispersive X-Ray spectrometers. The accelerating voltage was 15 kV, the current was 4 nA and the beam was 5 µm. On-peak count times were 30 s for Si, Al, P, Fe, Mn, Ca, Mg; 20s for Cl, Ti and K; and 10s for Na (and analysed first to minimise alkali loss). Background count times were 15s for Si, Al, P, Fe, Mn, Ca, Mg, Cl; 10 s for Ti and K; and 5s for Na. International secondary glass standards were run before, during and after the analysis to evaluate instrumental accuracy and precision. These standards include: ML3B-G (basalt), KL-2 (basalt), StHs6/80-G (andesite/dacite) and Lipari obsidian ID3506 (rhyolite) described in Kuehn et al. (2011) and Jochum et al. (2006). Glass standard analyses were within ±2% of published values (Online Resource 2: Table 1). For plotting, we excluded measurements with analytical totals < 96 wt. % and normalised data to 100 wt. % on an anhydrous basis.

Bulk rock major element concentrations were determined on a manually powdered sample at the Laboratoire G-Time of the Université libre de Bruxelles (Belgium). The sample was prepared as a solution by alkaline fusion. Loss on Ignition was estimated by heating the powder at 1000 °C for 2 hours after drying at 105°C for over 24h. Major elements were measured with a ThermoFisher Scientific iCAP Inductively Coupled Plasma - Optical Emission Spectrometer using Y as an internal standard. USGS standards BHVO-2, RGM-2 and QLO-1 were analysed as reference materials. Reproducibility based on reference materials was estimated to be generally better than 3% relative standard deviation (Online Resource 2: Table 2). Bulk rock trace elements

were measured at the University of Southampton. The sample was digested in HF/HNO_3 then analysed on an Agilent 8900 QQQ-ICP-MS calibrated with 10 international rock standards (Online Resource 2: Table 3).

References

Jochum KP, Stoll B, Herwig K, Willbold M, Hofmann AW, Amini M, Aarburg S, Abouchami W, Hellebrand E, Mocek B, Raczek I, Stracke A, Alard O, Bouman C, Becker S, Dücking M, Brätz H, Klemd R, de Bruin D, Canil D, Cornell D, de Hoog C-J, Dalpé C, Danyushevsky L, Eisenhauer A, Gao Y, Snow JE, Groschopf N, Günther D, Latkoczy C, Guillong M, Hauri EH, Höfer HE, Lahaye Y, Horz K, Jacob DE, Kasemann SA, Kent AJR, Ludwig T, Zack T, Mason PRD, Meixner A, Rosner M, Misawa K, Nash BP, Pfänder J, Premo WR, Sun WD, Tiepolo M, Vannucci R, Vennemann T, Wayne D, Woodhead JD (2006) MPI-DING reference glasses for in situ microanalysis: new reference values for element concentrations and isotope ratios. Geochemistry Geophysics Geosystems 7, Q02008. https://doi.org/10.1029/2005GC001060

Kuehn SC, Froese DG, Shane PAR, INTAV Intercomparison Participants (2011) The INTAV intercomparison of electron-beam microanalysis of glass by tephrochronology laboratories: results and recommendations. Quaternary International 246:19-47. https://doi.org/10.1016/j.quaint.2011.08.022
