# Supplementary figures and images for: The first documented volcanic eruption of Hayli Gubbi, Afar, Ethiopia
Source: Bull Volcanol. 2026 Jun 30;88(7):78. doi: 10.1007/s00445-026-01997-3 (PMC13319164; doi:10.1007/s00445-026-01997-3)

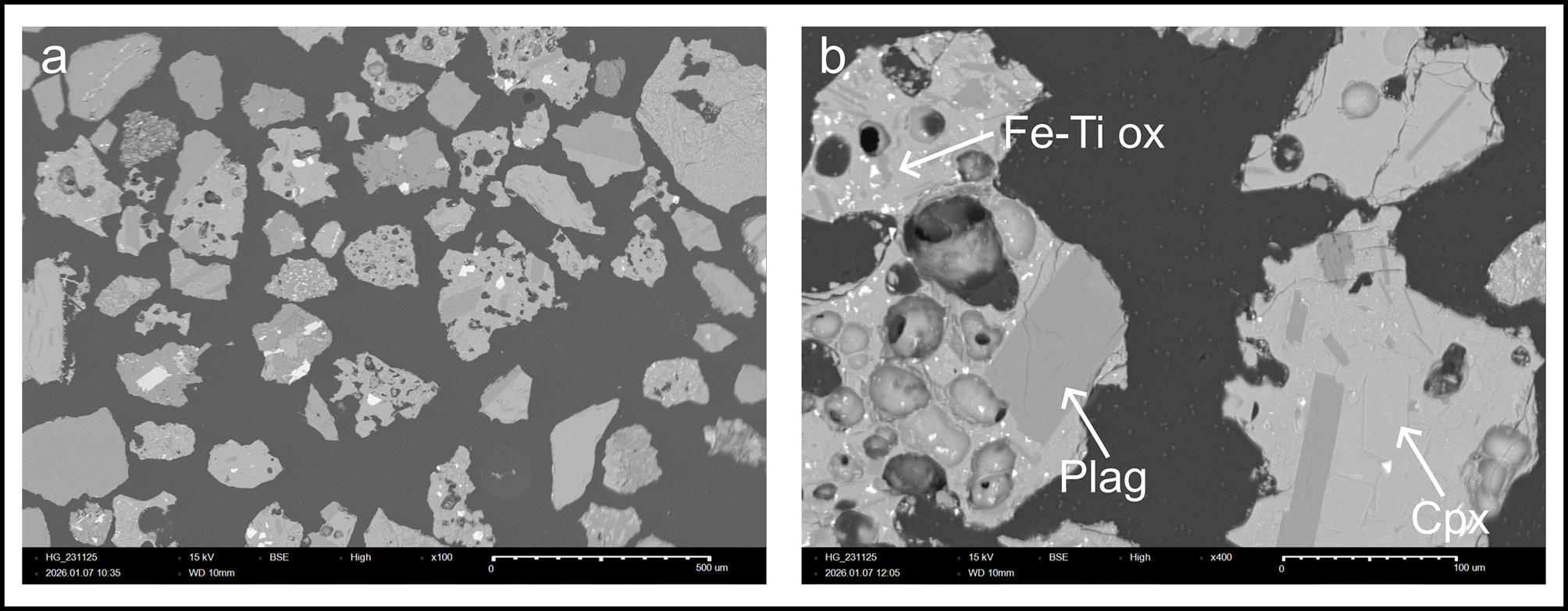

Supplement: Supplementary file 3 — (PNG 994 KB) [file 445_2026_1997_Fig9_ESM.png]

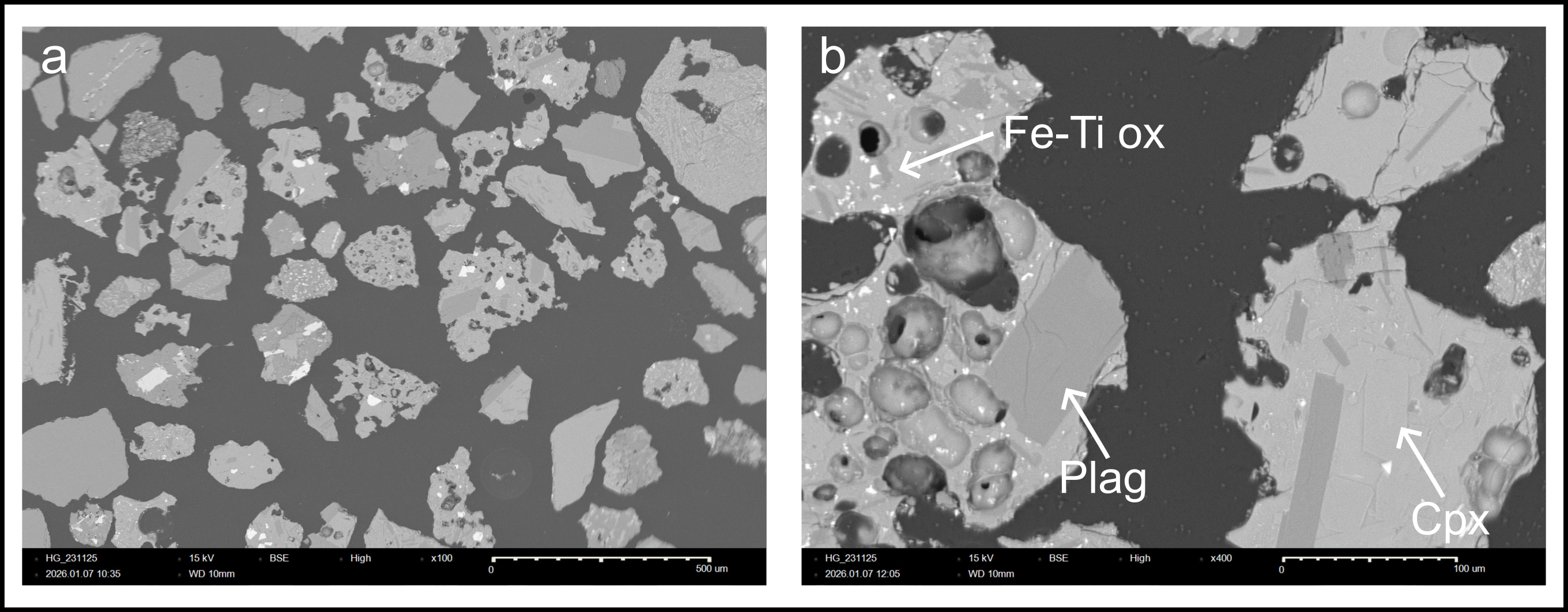

Supplement: Supplementary file 4 — (TIF.11.0 MB) [file 445_2026_1997_MOESM3_ESM.tiff]
